# Pre- and Postcopulatory Traits of* Salvator* Male Lizards in Allopatry and Sympatry

**DOI:** 10.1155/2016/8176267

**Published:** 2016-03-24

**Authors:** Sergio Naretto, Cecilia S. Blengini, Gabriela Cardozo, Margarita Chiaraviglio

**Affiliations:** Instituto de Diversidad y Ecología Animal (IDEA), CONICET and Laboratorio de Biología del Comportamiento, Facultad de Ciencias Exactas Físicas y Naturales, Universidad Nacional de Córdoba, Vélez Sársfield 299, X5000JJC Córdoba, Argentina

## Abstract

The reproductive traits of males are under influence of sexual pressures before and after copulation. The strength of sexual selection varies across populations because they undergo varying competition for mating opportunities. Besides intraspecific pressures, individuals seem to be subjected to pressures driven by interspecific interactions in sympatry. Lizards may vary their reproductive strategies through varying sexual characters, body size, gonadal investment, and sperm traits. We evaluated the reproductive traits, involved in pre- and postcopulatory competition, in allopatric and sympatric populations of* Salvator* lizards. We observed a spatial gradient of male competition among populations, with the following order: allopatric zone of* S. rufescens*; sympatric zone; and allopatric zone of* S. merianae*. Accordingly, variation in secondary sexual character, the relative testis mass, and the length of sperm component was observed between allopatry and sympatry in each species, suggesting differences in the investment of reproductive traits. However, we found that these two* Salvator* species did not differ in secondary sexual characters in sympatry. Interestingly, the trade-off between testes and muscle varied differently from allopatry to sympatry between these* Salvator* species, suggesting that the influence of social context on reproductive traits investment would affect lizard species differently.

## 1. Introduction

The reproductive traits of males are influenced by sexual selection before and after copulation [1, 2]. Precopulatory sexual selection may favor traits that are beneficial to males for access to mates (intrasexual competition), for improving mating success through mate choice (intersexual selection), or for both [3]. Furthermore, males may continue to compete after copulation through postcopulatory sexual selection, in particular sperm competition [4]. The potential strength of sexual selection varies greatly intraspecifically across populations [5]. Besides intraspecific pressures, individuals seem to be subjected to pressures driven by interspecific interactions when similar species are sympatric [6, 7]. The importance of precopulatory and postcopulatory pressures may vary and influence reproductive traits in relation to different social contexts.

When two ecologically similar species coincide in space and time, selective pressures can be generated in one or both of the species if they share the resources [8, 9]. The presence of hybrids indicate interspecific reproductive interaction; therefore, competition between species for the same mating resource may modify sexual selection on reproductive traits [9]. Furthermore, intensity of sexual selection depends critically on the availability of partners and competitors can also be influenced by the presence of related species [10, 11]. Intra- and interspecific interactions would represent a mixture of selective forces that provide an ideal scenario for studying evolutionary reproductive processes.

Variation in body size and robustness traits, secondary sexual characters, and gonad investment among male lizards may be related to differences in reproductive strategies [12]. Increased testicular mass may be associated with increased sperm production and be inferred as a strategy of copulation many times with a female or with many females. Moreover, body size is an important mate-quality signal in many species [1] and, in addition to the morphological traits of robustness, may benefit animals involved in agonistic interactions [2].For lizards, traits such as abdominal and tail perimeter might be important traits because they accumulate energetic resources there [13] improving mate search, fight, and copulation [14, 15]. Furthermore, an increased male head size may also be important in intersexual interactions [16–18] and intrasexual interactions [19]. Secondary sexual characters can determine the outcome of aggressive interactions and indicate reproductive condition or potential [20, 21]. In related species that used the same secondary sexual characters, sympatric individuals might experience reinforcement of male expression traits, whereas allopatric individuals do not, creating the potential for divergent sexual selection between sympatric and allopatric populations [22].

Postcopulatory sexual selection pressure on sperm traits may lead to differentiation between closely related species or populations of the same species [23]. Moreover, interpopulation differences in lizard genitalia associated to the presence of congenerics have been suggested [24]. Sperm competition has favored the evolution of larger testes [25] and drives the evolution of sperm traits that maximize the success of fertilization of males [26]. Several sperm traits have been proposed as determinant in the fertilization success, such as sperm concentration, because males need big number of spermatozoa to avoid sperm dilution inside female tract [27]. The size of different sperm components has been considered important in ejaculate quality by contributing differentially to diverse sperm functions [28, 29]. Furthermore, sperm velocity is known to be a major determinant of male fertility [30] 1999; [31, 32]. Carretero et al. [6] showed changes in sperm production in two lizard species in relation to social context, in which sympatric males produced more sperm than allopatric males. Hence, sperm competition may result in sperm traits dependent on social context [26].

*Salvator merianae* and* S. rufescens* (formerly* Tupinambis merianae* and* Tupinambis rufescens* [33]) provide an excellent model system because they are closely related species [34] and share bioecological traits [35]. The species are in allopatry in most part of their distribution area; however, they share a sympatric zone [35, 36]. In this sympatric area, reciprocal hybridization between these species and introgression by backcrossing occur [34]. Cabaña et al. [34] identified that hybrids occur only in the sympatry zone and are not randomly distributed across the study areas, as expected if they were the result of recent common ancestry, suggesting that* S. merianae* and* S. rufescens* have come into contact recently and this period would not have been enough to reach a degree of reproductive isolation between these species [34]. Moreover, agonistic interactions between males of both species during a copula event have been observed (personal observation) in sympatric zone; hence, they are competing for mates. Intrasexual competition may differ among species with different sex ratio of individuals qualified to mate [37] and can vary between populations of the same species [38]. In* S. merianae* and* S. rufescens* adult sex ratio is biased to males; however,* S. rufescens* have a more male-biased sex ratio than* S. merianae* indicating that the high intrasexual competition may be expected in* S. rufescens* [21]. Moreover, another indicator of intrasexual competition is sexual size dimorphism [39],* S. rufescens* being more dimorphic in sexual size than* S. merianae* [21]. In both species, the jaw muscle is a secondary sexual character. The increase in jaw muscle is associated with sperm presence and bigger testis size and can act as a sexual signal of reproductive condition [21]. In males of* S. merianae*, aggression and bite performance are crucial because more aggressive individuals are often better competitors for limited resources such as mates. Sperm traits showed substantial variation between species and among males within species [40]. Males of* S. merianae* present longer sperm than males of* S. rufescens*. However,* S. rufescens* presented higher sperm velocity than* S. merianae* males [40]. Finally, their breeding periods broadly overlap, in such way that gonad development, secondary sexual characters, and sperm presence are fully expressed during the same period [21, 29].

The aim of this study was to evaluate the reproductive traits, involved in pre- and postcopulatory competition, in allopatric and sympatric populations of* Salvator* lizards. In addition, we characterize the populations in relation to the availability of partners and competitors. If* S. rufescens* is a species subject to high intrasexual competition, we could expect that sympatric males do not increase their reproductive traits in comparison with allopatric males. While* S. merianae* in sympatry compete with a more competitive species, we expect that sympatric males should increase their reproductive traits compared to allopatry. In sympatry, interspecific interactions may cause convergence in traits involved in pre- and postcopula competition or divergence in reproductive strategies.

## 2. Materials and Methods

### 2.1. Species and Study Area

*Salvator merianae* and* S. rufescens* are similar in body size and exist in the southernmost area of genus distribution in South America [36, 41]. Both species are included in Appendix II of the Convention on International Trade of Endangered Species of Wild Fauna and Flora (CITES); in Argentina, commercial harvest is allowed [42] (Res. 11/2011, Secretaría de Ambiente y Desarrollo Sustentable de la Nación).

Sampling was conducted at three study sites in different social contexts: a zone of sympatry (30°54′W, 63°30′S to 31°10′W, 63°07′S) and two sites of allopatry (*S. merianae*: 31°28′W, 63°38′S to 31°45′W, 63°15′S;* S. rufescens*: 29°30′W, 64°15′S to 29°57′W, 63°55′S). The study sites were separated by a maximum distance of 100 km, exposed to very similar climatic conditions corresponding to similar biogeographic regions and under the same climatic isocline [29, 43] in order to minimize the effect of the environment. We sampled individuals from the same localities belonging to areas of sympatry and allopatry, as determined in Cardozo et al. [35] where Cabaña et al. [34] identified hybrids only in the sympatric zone.

Lizards were caught weekly from wild populations by local authorized hunters [42] during one season (2011) and only individuals during the reproductive period (October, November, and December) were used [21]. We are authorized for scientific capture by the government environmental agencies, and we selected and accompanied local authorized people with the aim of avoiding sex and size bias in capture rates. Specimens were killed for the legal skin trade, in accordance with AVMA Guidelines on Euthanasia [44]. The species were identified phenotypically on the basis of their coloration according to Cei [41].

### 2.2. Precopulatory Traits

We measured external dimorphic morphological traits [45]. In each specimen we recorded body mass (BM), snout vent length (SVL), abdominal perimeter (AP), and proximate tail perimeter (TP). We dissected and recorded superficial pterygoideus muscle mass (PMM) since it is secondary sexual trait [21] to the nearest 0.1 g using an electronic balance (OHAUS Traveler TA302).

### 2.3. Postcopulatory Traits

We recorded both testes mass (TM) to the nearest 0.1 g using an electronic balance (OHAUS Traveler TA302). To evaluate sperm concentration, spermatozoa were obtained from the terminal portion of the epididymis in plastic tube with phosphate buffered saline (PBS). Sperm concentration was estimated using a Neubauer chamber and the samples were diluted to a concentration of 1 × 10^6^ cells/mL in culture medium supplemented with 4% bovine serum albumin, prior to observation under a phase contrast microscopy Nikon eclipse Ti (Nikon Instruments Inc., Tokyo, Japan). Aliquots of sperm samples were fixed for photography in 2% formaldehyde [46] and stained with Blue Brilliant Coomassie. The samples were examined at 400x magnification under a phase contrast microscope Nikon eclipse Ti. Microphotographs were taken using Nikon DS-Qi1Mc digital camera with a DS-U2 controller. Absolute length (*μ*m) of head, midpiece, and flagellum and total sperm length of 50 spermatozoa per individual were measured using Image J software version 1.43u (NIH, Bethesda, MD). Then the ratio of flagellum : midpiece length was estimated. For sperm velocity aliquots (500 *μ*L) of sperm sample were incubated at 25°C for 30 min. Sperm velocity was measured using a video microscopy system (phase contrast microscope Olympus CX41 with a video camera ICAM 1500). The digital videos were captured with the Virtualdub v.1.6.16 software (Avery Lee). The samples were recorded at 100x magnification for 4 min. Subsequently, individual sperm tracks were followed for 3 s in 45 cells/sample and transformed to a matrix of Cartesian coordinates using ImageJ v.1.38 and its plug-in MtrackJ v. 1.1.0 (Eric Meijering). The straight line velocity (VSL; *μ*m/s) was calculated from this matrix using Spermtrack v. 4.2 (Universidad Nacional de Córdoba, Argentina) [29]. None of these variables showed association with body size (ANCOVA test: body size covariates were not significant).

### 2.4. Data Analyses

To determine availability of partners and competitors we calculated mature sex ratio during the reproductive period in each allopatric population studied. Additionally, we calculated a mature sex ratio of males and females considering both species in the sympatric zone, because the existence of reciprocal hybrids [34] indicates that females of both species could be a potential reproductive resource for males and interspecific agonistic interactions among males. To test differences in proportion of sexes we used Chi Square Test.

Prior to these analyses, we examined data for assumptions of normality and homogeneity of variance and variables were log-transformed when necessary. Two-factor ANOVAs were applied. When interactions between factors were significant, one-way ANOVAs were applied to each species separately to examine variation between the males of allopatric and sympatric populations. ANCOVAs were run to investigate variation in the characters (BM, AP, and TP), using SVL as a covariate. PMM and TM were analyzed with ANCOVA using BM as covariate. When ANCOVA was performed, the interaction of the factor with the covariate was evaluated. When the interaction was nonsignificant, it was discarded from the model. To obtain a measure of lizard's gonadal and muscle investment, we calculated residual scores from the general linear regression of log-transformed character to log-transformed body mass [47] for all populations. We then used these residuals as indices of investment. We compared the investment in testes and muscle using the relationship between these residuals. Statistical differences in the mean of sperm concentration and sperm morphometric and dynamic traits between populations were determined by Kruskal-Wallis or one-way nested ANOVA. Statistical analyses were performed using INFOSTAT version 2012 (Universidad Nacional de Córdoba) and SPSS 16.0 (SPSS 16.0 Inc., Chicago, IL, USA).

## 3. Results

Snout vent length did not differ between species and social context (ANOVA species term *F*_1,103_ = 0.31; *P* = 0.860; social context term *F*_1,103_ = 2.442; *P* = 0.121; species^*∗*^social context interaction *F*_1,103_ = 10.515; *P* = 0.002). However, in* Salvator merianae*, the SVL of mature males was greater in sympatry than in allopatry, whereas it was similar between populations in* S. rufescens* (Table 1) (ANOVA* S. merianae* social context term *F*_1,63_ = 14.589; *P* = 0.001; ANOVA* S. rufescens*: social context term *F*_1,40_ = 1.067; *P* = 0.308). Robustness characters of mature males (BM, AP, and TP) did not differ between species and social context and between these social contexts in each species (ANCOVA BM: species term *F*_1,98_ = 1.021; *P* = 0.315; social context term *F*_1,98_ = 0.120; *P* = 0.729; species^*∗*^social context interaction *F*_1,98_ = 0.100; *P* = 0.752; covariate term *F*_1,98_ = 380.238; *P* = 0.001; ANCOVA AP: species term *F*_1,98_ = 1.781; *P* = 0.185; social context term *F*_1,98_ = 0.101; *P* = 0.751; species^*∗*^social context interaction *F*_1,98_ = 2.328; *P* = 0.130; covariate term *F*_1,98_ = 118.630; *P* = 0.001; ANCOVA TP: species term *F*_1,97_ = 2.007; *P* = 0.160; social context term *F*_1,97_ = 0.009; *P* = 0.926; species^*∗*^social context interaction *F*_1,97_ = 0.296; *P* = 0.588; covariate term *F*_1,97_ = 212.659; *P* = 0.001).

Relative jaw muscle mass was greater in sympatry than in allopatry in* S. merianae* (*S. merianae* allopatry: PMM (mean ± SD) = 40.16 ± 26.03; *N* = 50; CV = 64.82;* S. merianae* sympatry: PMM (mean ± SD) = 78.10 ± 40.45; *N* = 14; CV = 51.79; ANCOVA social context term *F*_2,59_ = 5.594; *P* = 0.021; covariate term *F*_2,59_ = 269.608; *P* = 0.001). Conversely, in* S. rufescens* relative muscle mass was greater in allopatry than in sympatry (*S. rufescens* allopatry: PMM (mean ± SD) = 77.26 ± 44.76; *N* = 26; CV = 57.89;* S. rufescens* sympatry: PMM (mean ± SD) = 58.91 ± 46.18; *N* = 15; CV = 78.38; ANCOVA social context term *F*_2,37_ = 4.607; *P* = 0.038; covariate term *F*_2,37_ = 191.613; *P* = 0.001). Relative testis mass was greater in sympatry than in allopatry for* S. rufescens* (*S. rufescens* allopatry: TM (mean ± SD) = 6.13 ± 3.59; *N* = 25; CV = 58.52;* S. rufescens* sympatry: TM (mean ± SD) = 8.56 ± 4.90; *N* = 15; CV = 57.24; ANCOVA social context term *F*_2,37_ = 6.075; *P* = 0.019; covariate term *F*_2,37_ = 18.877; *P* = 0.001) but this parameter did not differ between social contexts in* S. merianae* (*S. merianae* allopatry: TM (mean ± SD) = 4.10 ± 2.54; *N* = 51; CV = 61.88;* S. merianae* sympatry: TM (mean ± SD) = 5.84 ± 3.99; *N* = 14; CV = 68.36; ANCOVA social context term *F*_2,59_ = 0.080; *P* = 0.779; covariate term *F*_2,59_ = 17.048; *P* = 0.001).

The comparison of sperm traits in* S. merianae* showed differences in sperm length, with sperm with longer midpiece length in sympatry than in allopatry. Hence, the flagellum : midpiece ratio was shorter in the sympatric than in the allopatric population. However, sperm concentration and VSL were similar between social contexts (Table 2). Although in* S. merianae* there are no significant differences between social contexts in total sperm length, there is a tendency to a decrease in sperm size in sympatry (Table 2). On the other hand,* S. rufescens* did not present differences in any of the measured sperm traits between populations (Table 2).

Pterygoideus muscle mass and SVL were similar between males of* S. merianae* and* S. rufescens* in sympatry, whereas there were differences between species in relative testis mass and sperm morphometric traits.* Salvator rufescens* had bigger testes than* S. merianae*. Furthermore,* S. merianae* presented longer midpiece and longer flagellum length than* S. rufescens* (Table 3).

**Figure 1:**
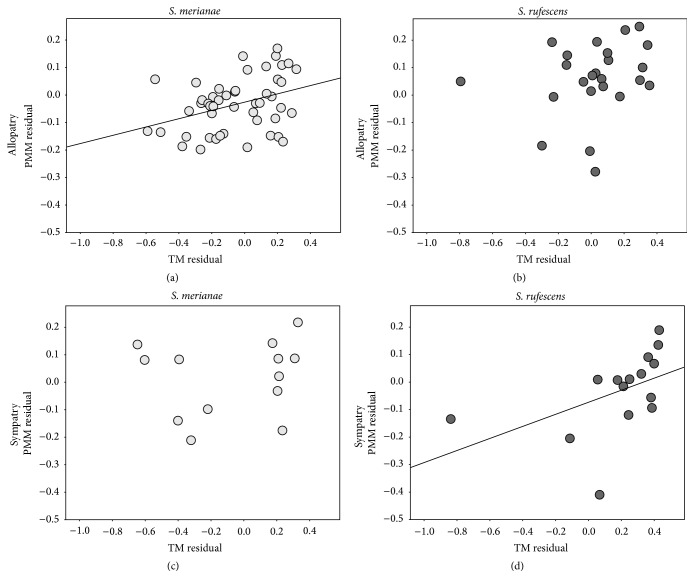
Relationship between testes investment and muscle investment for (a)* Salvator merianae* in allopatry, (b)* S. rufescens* in allopatry, (c)* S. merianae* in sympatry, and (d)* S. rufescens* in sympatry.

We observed a positive relationship between pterygoideus muscle investment and testes investment in* S. merianae* in allopatry (*F*_1,46_ = 6.866, *R* = 0.13, *P* = 0.012) but not in sympatry (*F*_1,12_ = 0.72, *R* = 0.06, *P* = 0.793). In* S. rufescens,* we observed a positive relationship in sympatry (*F*_1,13_ = 3.895, *R* = 0.23, *P* = 0.05) but not in allopatry (*F*_1,22_ = 1.319, *R* = 0.06, *P* = 0.263) (Figure 1).

Mature sex ratio during the reproductive period of* S. merianae* in allopatry was 1.59 : 1 (Chi Square Test: *χ* ^2^ = 4.39; d.f. = 1; *P* = 0.036) and in sympatry was 1.55 : 1 (Chi Square Test: *χ* ^2^ = 1.10; d.f. = 1; *P* = 0.295). Mature sex ratio of* S. rufescens *in allopatry was 6.75 : 1 (Chi Square Test: *χ* ^2^ = 19.13; d.f. = 1; *P* < 0.001) and in sympatry it was 2.50 : 1 (Chi Square Test: *χ* ^2^ = 3.98; d.f. = 1; *P* = 0.045). Mature sex ratio was different between species (Chi Square Test: *χ* ^2^ = 6.37; d.f. = 1; *P* = 0.018) but was similar between social condition in each species (*S. merianae*: Chi Square Test: *χ* ^2^= 0.025; d.f. = 1; *P* = 0.966;* S. rufescens*: Chi Square Test: *χ* ^2^= 1.94; d.f. = 1; *P* = 0.163). Mature sex ratio of* Salvator* lizards in sympatry (considering both species) was 1.93 : 1 (Chi Square Test: *χ* ^2^ = 4.53; d.f. = 1; *P* = 0.033), being different from allopatric zones (Chi Square Test: *χ* ^2^ = 7.77; d.f. = 2; *P* = 0.020).

## 4. Discussion

Populations of* Salvator merianae* and* S. rufescens* differ in some reproductive traits between social contexts, suggesting that they might be subjected to different selective pressure caused by the presence of related species competing for the same resources. Not only the presence of other species but also the relative availability of mate and competitors could influence reproductive strategies. In both populations of* S. rufescens*, mature sex ratio during the reproductive period was biased to males. By contrast, in* S. merianae*, mature sex ratio during the reproductive period was biased towards males only in allopatry but not in sympatry. However, mature sex ratio presents similar intensity in different social contexts in* S. merianae* and with lower bias than* S. rufescens*. Considering sex ratio by zone, we observed a gradient from high to low competition between males for mating opportunities, with the following order: allopatric zone of* S. rufescens*; sympatric zone; and allopatric zone of* S. merianae*. In fact, if we consider that interspecific sexual interactions allow getting offspring [48], males of* S. merianae* in sympatry could afford higher relative competition for mating opportunities than males of* S. merianae* in allopatry, whereas males of* S. rufescens* could exhibit an opposite pattern. These results pose scenarios that allow more fully interpreting the strategies at different levels of reproductive competition.

Variation in the relative pterygoideus muscle mass, relative testis mass, and length of sperm component was observed between populations, suggesting differences in the investment of traits for reproduction. However, robustness characters of mature males were similar between social contexts in both species. Hence, these jaw muscles, testes, and sperm traits vary as a result of differential expression of traits and not due to allometry. Different scenarios of reproductive competition can influence the intensity of pre- and postcopulatory pressure. In* S. rufescens*, the intensity of competition for mating opportunities was higher in allopatry than in sympatry; hence, males exhibited higher PMM in this allopatric population, suggesting that muscle could be an important functional character to afford a strong intrasexual precopula competition, in fighting or as a sexual signal. Moreover, relative female availability (conspecific or heterospecific) was higher for* S. rufescens* in sympatry, which exhibited greater relative testis size and smaller pterygoideus muscle mass than males in allopatry. While differences were not expressed in sperm concentration, testicular enlargement may be related to increased fluid ejaculate quantity as a response to access to many females [49] avoiding depletion effect [50]. Females can copulate with different males, even on the same day [51]. Follicular development is only completed several days after mating; it would also require sperm retention in female genital ducts [52]. Competition for fertilization is important because females have once-a-year clutch production with high number of eggs [13, 40, 50]. Testicular increase has also been reported as an indicator of risk of sperm competition [25] and* S. rufescens* in sympatry may experience higher postcopula competition than in allopatry. However, sperm traits were not different between populations of* S. rufescens*. In turn, in* S. merianae* availability of conspecific mate resource did not differ between populations. However, in sympatry the presence of males of* S. rufescens* increased the relative number of competitors for males of* S. merianae*. Regarding precopulatory competition, males of* T merianae* in sympatry had greater SVL and more development of secondary sexual character, which could be explained by competition with males of both species [8, 53]. We found that* S. merianae* in sympatry produced sperm with longer midpiece length than in allopatry. An elongation of the midpiece may result in stronger propulsion force, more mitochondria, and an increase in the energy produced by glycolysis [46] that may improve sperm longevity. The midpiece could be the main component providing sperm with energy to survive in the female tract. Accordingly, the increase in midpiece in sympatry suggests an additional investment in sperm quality by* S. merianae*.

Reinforcement is the process of selection against mating or hybridization with closely related taxa [54]. We found that these two* Salvator* species did not differ in secondary sexual characters and morphological robustness characters in sympatry. Not only do males fight, but also they also display courtship where females can reject males [51]. During copulation the male lizard typically bites the female on the back of the neck as he mounts her [21] but females are able to escape from males [51]. Within a clade, males of one species or population could express the same secondary sex trait as females use in mate choice [55]. These similarities in traits might imply that the same forces of evolution that influence the signal of sexual quality used in one species also influence the signal that makes a mate more attractive or a better competitor in other closely related species. In the sympatric zone we observed that sperm concentration, total sperm length, and VSL also are similar between species. The similarities in traits involved in pre- and postcopulatory traits suggest that these species are in competition by resource with same weapons and that reproductive isolation barriers in these lizards are relaxed. This might explain reciprocal hybridization between* S. merianae* and* S. rufescens* observed [34]. Even though, another explanation of convergence in traits is that hybridization can yield a very similar pattern [9], but we show in this paper variability of reproductive traits in* Salvator* lizards under different social contexts in wildlife, and it is difficult to determine their causes. Both divergence and convergence of different traits in sympatry are possible outcomes, depending on the intensity of interspecific competition [9] and species strategies.

The influence of social context on decisions in reproductive character investment would affect lizard species differently; hence, we postulate that reproductive investments in traits are not fixed. Moreover, there is growing evidence to suggest that males face a trade-off between the allocation of resources to precopulatory competition for access to females and postcopulatory competition to fertilize eggs [10], mainly in insects, birds, and fishes (males with large weapons have relatively smaller testes and smaller ejaculate volumes than males with small weapons). Interestingly, the trade-off between testes and muscle varied differently from allopatry to sympatry between these* Salvator* species. The pattern observed could be explained by the presence of both conspecific and heterospecific rivals when both species coexist, the difference in availability of mates, and a combination of both. We cannot discriminate between the selective forces over pre- and postcopulatory traits modulating the reproductive strategies.

## 5. Conclusions

Evolutionary shifts in the same traits could arise through different mechanisms and yield the same effects on reproductive strategies. Clearly, multiple processes could contribute to any given phenotypic shift and separating them empirically can be difficult; however, in this study we showed that social context influences reproductive traits, suggesting a trade-off between pre- and postcopulatory traits in allopatric and sympatric populations of* Salvator* lizards.

## Figures and Tables

**Table 1:** Descriptive statistics of body size and robustness parameters for mature males during the reproductive period of the studied populations (allopatry and sympatry) in each species.

	*Salvator merianae*		*Salvator rufescens*	
Character	Allopatry	Sympatry	Allopatry	Sympatry
Snout vent length (cm)	38.86 ± 3.17^a^	42.46 ± 2.93	41.42 ± 3.47	40.17 ± 4.30
	51^b^	14	27	15
	8.17^c^	6.89	8.39	10.72

Body mass (g)	1961 ± 544	2539 ± 608	2388 ± 756	2243 ± 728
	49	14	25	15
	27.73	23.94	31.66	32.45

Abdominal perimeter (cm)	24.42 ± 3.24	26.07 ± 4.10	26.06 ± 4.02	25.87 ± 3.28
	49	14	25	15
	13.29	15.74	15.41	12.66

Tail perimeter (cm)	16.06 ± 1.56	17.38 ± 1.21	17.20 ± 1.97	16.83 ± 2.09
	49	13	25	15
	9.69	6.96	11.42	12.43

^a^Mean ± SE, ^b^sample size, and ^c^CV.

**Table 2:** Comparison of sperm parameters between social contexts in *S. merianae* and *S. rufescens*.

Sperm traits	Males in allopatry	Males in sympatry	Statistic	*P* value
*Salvator merianae*				
Concentration (10^6^ cell/mL)	2204.2 ± 942.50	2750.63 ± 292.73	*H* = 1.18	0.2773
	*N* = 13	*N* = 8		
	42.76	10.64		
Head length (*μ*m)	14.07 ± 1.53	13.0 ± 1.13	*F* = 3.28	0.0846
	*N* = 14	*N* = 9		
	10.91	8.71		
Midpiece length (*μ*m)	5.08 ± 0.21	5.29 ± 0.21	**F** = 5.49	**0.0291**
	*N* = 14	*N* = 9		
	4.08	4.04		
Flagellum length (*μ*m)	59.88 ± 0.77	59.16 ± 1.76	*F* = 1.84	0.1894
	*N* = 14	*N* = 9		
	1.29	2.97		
Total sperm length (*μ*m)	79.7 ± 1.64	78.07 ± 2.63	*F* = 3.36	0.0808
	*N* = 14	*N* = 9		
	2.06	3.36		
Flagellum/midpiece ratio	11.9 ± 0.44	11.29 ± 0.49	**F** = 9.60	**0.0054**
	*N* = 14	*N* = 9		
	3.68	4.33		
Straight line velocity (*μ*m/s)	28.79 ± 6.23	29.37 ± 6.36	*F* = 0.05	0.8317
	*N* = 14	*N* = 8		
	21.64	21.66		
*Salvator rufescens*				
Concentration (10^6^ cell/mL)	2289.69 ± 934.18	2621.67 ± 1103.62	*H* = 0.39	0.5334
	*N* = 16	*N* = 9		
	40.80	42.10		
Head length (*μ*m)	13.09 ± 1.47	13.11 ± 1.50	*F* = 0.0011	0.974
	*N* = 18	*N* = 9		
	11.24	11.45		
Midpiece length (*μ*m)	4.96 ± 0.16	4.92 ± 0.19	*F* = 0.24	0.6277
	*N* = 18	*N* = 9		
	3.19	3.81		
Flagellum length (*μ*m)	57.42 ± 1.09	57.3 ± 1.09	*F* = 0.07	0.7911
	*N* = 18	*N* = 9		
	1.90	1.91		
Total sperm length (*µ*m)	76.21 ± 1.90	75.94 ± 2.51	*F* = 0.12	0.7326
	*N* = 18	*N* = 9		
	2.49	3.30		
Flagellum/midpiece ratio	11.74 ± 0.41	11.81 ± 0.48	*F* = 0.16	0.6915
	*N* = 18	*N* = 9		
	3.46	4.08		
Straight line velocity (*μ*m/s)	30.09 ± 7.91	27.83 ± 408	*F* = 0.68	0.4175
	*N* = 18	*N* = 9		
	26.27	14.67		

**Table 3:** Comparison between species in sympatry.

Traits	d.f.	*F*	*P* value
SVL (cm)	1.27	2.785	0.1067
PMM	1.26	0.104	0.7501^a^
		70.765	0.0001^b^
TM	1.26	5.83	**0.0231**
		7.97	0.009
Sperm concentration (10^6^ cell/mL)	1.16	*H* = 0.15	0.743
Head length (*μ*m)	1.16	*F* = 0.03	0.8602
Midpiece length (*μ*m)	1.16	*F* = 15.05	**0.0013**
Flagellum length (*μ*m)	1.16	*F* = 7.26	**0.016**
Total sperm length (*μ*m)	1.16	*F* = 3.10	0.0974
Flagellum/midpiece ratio	1.16	*F* = 5.09	**0.0385**
Straight line velocity (*μ*m/s)	1.15	*F* = 0.40	0.5354

^a^Species effect, ^b^covariate effect.
